# Epididymal approaches to male contraception

**DOI:** 10.1186/s12610-018-0078-y

**Published:** 2018-11-06

**Authors:** Joël R. Drevet

**Affiliations:** Laboratoire GReD “Génétique, Reproduction & Développement”, UMR CNRS 6293, INSERM U1103, Université Clermont Auvergne (UCA), 28-Place Henri Dunant, bâtiment CRBC, 63000 Clermont-Ferrand, France

**Keywords:** Spermatozoa, Post-testicular sperm maturation, Non-hormonal contraception, Spermatozoïdes, maturation post-testiculaire, contraception non-hormonale

## Abstract

Today, a vast arsenal of contraceptive methods interfering at different levels of the female reproductive axis is available. This is not the case for men for whom, until now, there is no reliable male reversible method and for whom vasectomy, condom and withdrawal are the only options available. Despite this limited supply, more than one third of all contraceptive methods used worldwide rely on the cooperation of the male partner. Besides developing hormonal approaches to stop sperm production, there may be attractive approaches that will interfere with sperm functions rather than production. Sperm functions are primarily established during post-testicular maturation, with the epididymis accounting for the majority. The purpose of this review is to present some of the promising and/or already abandoned leads that emerge from research efforts targeting the epididymis and its activities as potential means to achieve male post-meiotic contraception.

## Résumé

Aujourd’hui, un vaste arsenal de méthodes contraceptives interférant à différents niveaux de l’axe reproductif féminin est. disponible. Ce n’est. pas le cas des hommes pour qui, jusqu’à présent, il n’existe pas de méthode masculine réversible fiable et pour qui la vasectomie, le préservatif et le retrait sont les seules options à disposition. Malgré cette offre limitée, plus d’un tiers de toutes les méthodes contraceptives utilisées dans le monde entier reposent sur la coopération du partenaire masculin. A côté du développement d’approches hormonales pour arrêter la production de sperme, il peut y avoir des approches attrayantes qui interféreront avec les fonctions du sperme plutôt qu’avec la production. Les fonctions des spermatozoïdes sont principalement établies pendant la maturation post-testiculaire, l’épididyme assurant la plus grande part. Le but de cette revue est. de présenter certaines des pistes prometteuses ou/et déjà abandonnées qui ressortent des efforts de recherche ciblant l’épididyme et ses activités comme moyens potentiels de parvenir à une contraception post-méiotique masculine.

Despite the range of contraceptive methods available, 38% of pregnancies worldwide are unwanted and 22% end in abortion, clearly suggesting the need for a wider choice of contraceptive methods. Until today, fertility pharmacological control methods that offer a good level of safety and efficacy and are easy to implement concern only women [1]. As far as men are concerned, the supply of contraceptive techniques is much more limited (condoms, vasectomy and “coitus interruptus”) and there is still no reversible male pharmacological contraceptive on the market. Yet about one-third of all contraceptive methods used worldwide rely on the “cooperation” of the male partner. With the new possibilities brought about by the era of molecular biology, there is now a chance that pharmacological means of controlling male fertility can be developed and that the range of choices available to men can be expanded so that they can play a greater part in regulating their fertility [2, 3].

**Fig. 1:**
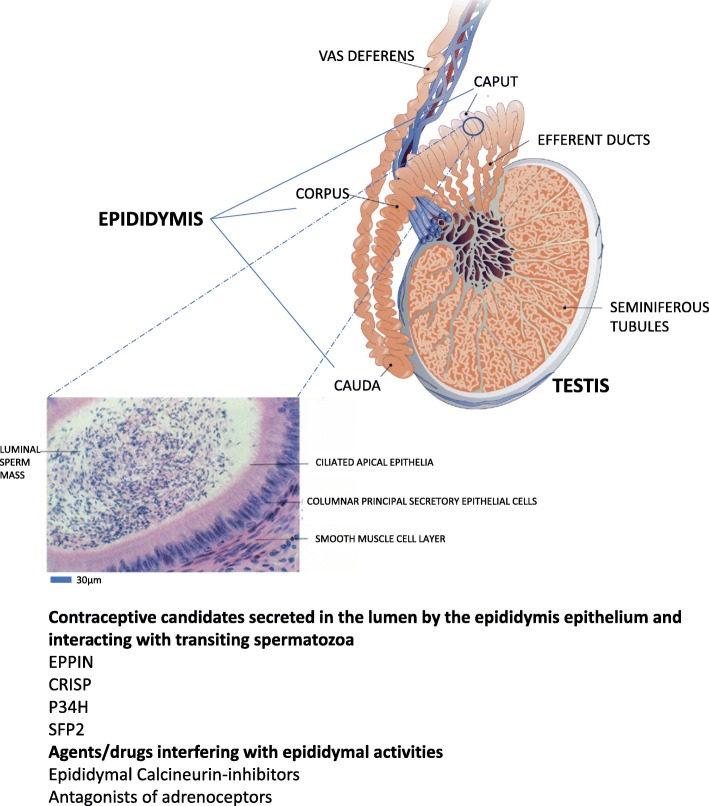
Schematic representation of the anatomical organization of the mammalian epididymis connecting the testicular seminiferous tubules through the efferent ducts to the vas deferens. A magnification of an epididymal tubule is shown pointing to the epididymal epithelial layer and luminal compartment in which sperm progress through the peristaltic contractions of the smooth muscle layer surrounding the tubule. The liquid luminal content critical for post-testicular acquisition of sperm fertilization capabilities is the result of intense and complex secretory activities of the epididymal epithelium, some of which could be targeted for the development of a post-testicular contraceptive agent

## The epididymis and its functions: Avenues for the development of new contraceptive strategies

In summary, there are 3 possible pharmacological approaches to male contraception: 1) interfere with the production of male gametes in the testis, 2) interfere with the post-testicular acquisition of the fertilizing capacities of spermatozoa, in other words, interfere with the functions of the epididymis since it is in this tubule that spermatozoa acquire their fertilizing power and are prepared for the ultimate events that precede fertilization: capacitation and acrosomal response (for a review on the epididymis and its functions see: [4], and finally, 3) interfere with mature gametes directly.

How is the epididymis and its associated functions interesting for developing new post-test contraceptive strategies is detailed below?

Spermatozoa produced within the male gonad leave the seminal epithelium via the *rete testis* and efferent ducts to enter the epididymal tubule. Thanks to the peristaltic contractions of the smooth muscles surrounding the epididymal tubule and epididymal fluid flow, the gametes progress towards the terminal part of the organ, the tail of the epididymis or cauda and their storage place between two ejaculations. This trip for most mammals takes about 10 days. Although the gametes that enter the epididymis appear structurally completely differentiated, they are functionally immature. This immaturity is characterized by their inability to move and to recognize and penetrate an egg. These functional parameters (mobility, ability to fertilize) are gradually acquired during the epididymal descent [5]. At the end of epididymal maturation, cauda epididymal spermatozoa are functionally competent and able to fertilize an egg. Given the silent nature of spermatozoa after spermatogenesis, i.e. the absence of cell-autonomous transcription and translation events, all the changes they undergo during descent into the epididymal tubule are due to the activities of the epididymal fluid and, by extrapolation, to the activities of the epididymal secretory epithelium.

Briefly, all spermatozoa regions (head, intermediate piece, flagella), all compartments (acrosome, nucleus, mitochondrial spindle, ...) and all constituents (proteins, lipids, carbohydrates, nucleic acids) will be concerned by the events of epididymal maturation. Although we are still far from knowing in detail all the changes that accompany this epididymal maturation of spermatozoa, their sequence as well as the functional consequences of these changes on gametes, we are beginning to have a fairly clear vision of certain aspects of this maturation (for a review see: [6]).

For example, it is clear that during epididymal transit the profile of the surface proteins of the male gamete but also of the internal proteins is modified. This involves the acquisition of new proteins derived from merocrine secretion processes of the epididymal epithelium but also, by the transfer of proteins that do not possess a secretory signal peptide via lipid vesicles (called epididymosomes) resulting from apocrine secretion processes [7]. This also involves more subtle modification events of proteins acquired de novo and/or already present on gametes via proteolysis, differential glycosylation/deglycosylation events and all other types of post-translational protein modifications (sulfoxidation, phosphorylation, sulfatation, sumoylation....). The lipid profiles of the gametes are also profoundly altered during the epididymal descent of the gametes by processes that are little known to date but which ultimately confer to this cell particular membrane properties in terms of fluidity, “raft and non-raft” domains sequestering cell signaling actors involved in triggering capacitation and acrosomal reaction (for reviews see: [8, 9]).

Another aspect of epididymal sperm maturation that needs to be addressed concerns the protection and survival of gametes in transit and stored in the terminal part of the tubule. As already mentioned above, post-testicular spermatozoa are silent cells that have little or no ability to defend themselves against attacks to which they may be subjected. Indeed, these cells cannot mount transcriptional and translational responses to any stress, nor can they count on the protection that their cytoplasmic enzymatic equipment can provide, since they have evacuated most of their residual cytoplasm upon spermiation in the testis. The epididymis and epididymal fluid therefore ensure, via different activities, the protection of these cells during their transit and during storage periods between two ejaculations. An important aspect of this epididymal protection of spermatozoa concerns the antioxidant capacities of the epididymal territory which control both the proper maturation of sperm cells and also the extent of oxidative damage to the spermatozoa. From the last two decades, it became obvious that sperm oxidative alterations constitute an important part of male infertility by affecting, among other things, spermatozoa mobility and the integrity of the paternal chromosomal lot (for a review see: [10]).

Understanding all aspects of this post-testicular maturation of male gametes is a challenge not only for the diagnosis and possible therapy of male infertility with normal spermatogenesis (which represents half of male infertility cases) but also for what concerns us here, i.e. the development of new post-testicular contraceptive strategies. Indeed, the idea has logically emerged that reversibly interfering with one or more of these epididymal activities could be used for contraceptive purposes [11–18]. The examination of the physiology of epididymal function has received little attention over the years, and even today, still receives minimal attention, however the approaches developed in the last 10 years by a small number of research groups have made it possible to apprehend the multiplicity and complexity of events in the epididymal maturation of spermatozoa. As has been the case for many other tissues, the epididymis has benefited from recent large-scale exploration techniques (transcriptomics and proteomics) which have made it possible to identify genes and proteins expressed in a particular way in this territory [19–31]. These approaches generated large quantities of results highlighting genes and proteins with known functions but also genes and proteins that were not suspected to be expressed in the epididymis. The problem in this profusion of findings is now to validate the function and importance of these genes and proteins in epididymal maturation and in male fertility and, to select which might prove to be attractive contraceptive targets. In practice, “interesting” means proteins or activities that can be targeted pharmacologically. It is at this level that mutant animal models reveal their power by specifically assessing the reproductive impact of the invalidation of a given gene and thus its potential as a contraceptive pathway.

Conceptually, targeting the epididymis and its functions for contraceptive purposes may appear attractive in at least three ways that respond in part to the limitations of hormonal strategies aimed at blocking the production of gametes in the testis [32]. The first advantage is not to disturb the spermatogenesis and to act only on the functional parameters of the gametes resulting from the testis. The second advantage, on paper at least, concerns the speed of action as well as the speed of reversibility of action. Indeed, spermatogenesis is a slow process which in men covers about 10 weeks. Spermatozoa then pass through the epididymis for about 10 days and are stored for a time that will depend on the sexual activity of the individual. An epididymal fertility control agent should not disturb spermatogenesis, and act faster than an agent that affects testicular function. The third advantage is that given the multiplicity of gamete changes during epididymal descent, it may be possible to find a strategy (an agent) that does not involve the hormonal component. Considering the pleiotropic effects played by hormones on physiological processes outside gametogenesis, it would thus be possible in absolute terms to reduce the side effects inherent in taking hormonal contraceptives. As is also the case for testicular targets, the presence of a blood/epididymal barrier (BEB) poses a problem with regard to the administration modalities (which ideally should be the oral route) and, most importantly, to the effectiveness of a possible epididymal contraceptive agent. Both the BTB (Blood Testis Barrier) and the BEB are there to create a sealed luminal testicular and epididymal environment mainly to establish a situation of immune privilege so that spermatic antigens do not switch on the adaptative immune response, a situation that would be detrimental to sperm cells and fertility. Several adaptations of the junctional system of the concerned epithelia plus complex immune suppressive processes have been evolved to respond to this situation known as “peripheral tolerance” [33, 34]. The consequences are that it renders the luminal compartments of the tubules (seminiferous and epididymal) less accessible to molecules coming from the interstitial compartment (ie: the blood) which poses great limitations in terms of drug/agent bioavailability within these tubules where they are expected to exert their contraceptive actions. However, recent data suggest that the BEB appears to be a lot less solid than the BTB theoretically offering greater permeability [34].

Targeting the epididymis and its functions could thus meet at least 2 of the 5 essential criteria put forward by manufacturers wishing to optimize the male contraceptive offer: speed of action and safety. However, it remains to choose the right targets to meet the other three criteria: effectiveness, reversibility and ease of use.

The elements that follow do not claim to be exhaustive and present all the epididymal genes and proteins that could prove to be potentially interesting in a contraceptive aim (as summarized in Fig. 1). I will limit my remarks to a few convincing and/or promising examples that illustrate that the epididymis and its functions could allow new, non-hormonal contraceptive approaches which is not yet a reality. I will also mention some leads that have now been abandoned.

Although there have been early attempts at epididymal contraception in animal models based on direct injection of metal compounds (Copper, Zinc and various derivatives) into the tail of the epididymis, no summary of these experiments will be made here (for an example see: [35–37]). These attempts led in some cases to reversible infertility, often accompanied by tissue alterations of the epididymis and/or the testis, associated with germline apoptosis. The toxicity induced by these strategies does not make them interesting clinical leads. Some attempts to interfere with major epididymal secretions have also been tested without great success, for example with the use of the antibiotic pivampicillin which promotes urinary excretion of carnitine or the use of catanospermine, a neutral glucosidase inhibitor [38]. For the latter strategies, even if the fertility of the treated animals could be reduced, this never led to reversible sterility.

## Proteins and epididymal activities in the pipeline of potential post-testicular contraceptives

### Eppin’s case: the most promising lead

The Reproductive Biology Laboratory at Chapel Hill (North Carolina, USA) in collaboration with the Human Genome Sciences program (Rockville, Maryland, USA) generated human epididymis cDNA libraries [39] for the purpose of obtaining epididymis-specific gene sequences. Among the hundreds of cDNA clones obtained, a cDNA coding potentially for an epididymis-specific protease inhibitor not yet identified has been selected. The clone has been called EPPIN for “EPididymal Protease INhibitor” [40] and is also known generically as SPINLW1. The corresponding gene has been identified and its three messenger RNA products code for two isoforms of a protein rich in cysteine residues having both a KUNITZ-type domain and a “WAP-type 4-DSC” domain; classical domains of protease inhibitors [40]. Two of the EPPIN isoforms (EPPIN-1 and EPPIN-3) show a peptide signal of secretion. In humans, the EPPIN gene is located on chromosome 20 in position 20q12–13.2 [40]. Genetic polymorphisms of EPPIN have recently been reported, some associated with infertility [41]. Although predominantly epididymal in expression, a more detailed transcriptomic study revealed that EPPIN is not strictly epididymis-specific since the testis (Sertoli cells) also expresses and secretes EPPIN which is thus found in small proportion on the surface of testicular spermatozoa. In the efferent ducts and in the epididymis the isoform EPPIN-1 is secreted by epithelial cells and is found both on the surface of spermatozoa and on the apical edge of epididymal epithelial cells. In these tissues, the expression of EPPIN-1 was shown to be controlled by androgens [42–44].

The function(s) of EPPIN began to emerge when it appeared: 1) that EPPIN had the ability to bind to semenogelin (SEMG1) a protein secreted by seminal vesicles, 2) that EPPIN logically possessed antimicrobial activity for a protease inhibitor [45, 46] and finally, 3) EPPIN modulated the serine protease activity of PSA (Protate Specific Antigen). Indeed, it has been shown that EPPIN modulates semenogelin hydrolysis by PSA and that in the absence of EPPIN, PSA hydrolyses semenogelin into small peptides [47]. Conversely, in the presence of EPPIN on the surface of gametes, semenogelin is partially protected from hydrolysis by PSA [48]. How EPPIN attaches itself to the gamete has also been elucidated. EPPIN was found on the surface of gametes in a protein complex combining clusterin (CLU) and lactotransferrin (LTF) [49] distributed foci along the main part of the flagellar axis. EPPIN does not have its own receptor but it is hypothesized that the LTF and CLU receptors contribute to stabilize EPPIN in the complex at the surface of the gametes. At ejaculation, sperm leave the epididymis, mix with seminal vesicle secretions and semenogelin is added to the EPPIN/LTF/CLU complex. The fixation of semenogelin to EPPIN blocks the progressive rectilinear mobility of gametes [50]. When prostatic fluid is added to the ejaculate, PSA hydrolyses semenogelin during the liquefaction phase, thereby releasing rectilinear progressive mobility [51].

The importance of EPPIN in reproductive function was tested by an immunological approach in non-human primates (*Macaca radiata*) rather than by developing a knockout mouse model because semenogelin is not expressed in mice. Several male monkeys (5 out of 9) that showed a high titer of anti-EPPIN antibody after immunization were found infertile [52] clearly suggesting that EPPIN is an important protein for reproductive function. In these immune animals, sperm mobility and the ability of EPPIN to bind semenogelin were affected by anti-EPPIN antibodies. Two dominant epitopes responsible for the contraceptive effect of anti-EPPIN antibodies have been identified respectively in the N and C-terminal domains of the protein [53]. Most recently, an antibody specifically directed against the epitope of the C-terminal domain has shown a powerful inhibitory effect on sperm mobility in humans [51].

Thus, immunization with an anti-EPPIN antibody results in effective and reversible contraception which passes through blocking of the semenogelin binding site on EPPIN inducing a progressive rectilinear loss of gamete motility. With this proof of concept established, the next step was to look for organic compounds that could have the same effect as the anti-EPPIN antibody, i.e. block the semenogelin binding site and inhibit sperm mobility. Screening has been performed to isolate compounds that have the in vitro ability to prevent binding of the anti-EPPIN antibody [51]. Approximately 100,000 compounds have been tested by a high throughput approach for their ability to inhibit sperm mobility [53]. Some compounds have been shown to be effective and are presently under study. A promising EPPIN-based lead contraceptive compound (EP055) showing in vivo contraceptive effects in monkeys was reported [54] that could provide a reversible, short-lived pharmacological alternative.

### The Cystein-rich family of secreted proteins (CRISP)

The CRISP (cysteine-rich sperm proteins) family of mammals has 4 members: CRISP1 (also called DE protein or AEG), CRISP2 (also called TPX1), CRISP3 and CRISP4. In mice, only CRISP1 and CRISP4 are expressed in the epididymis [55, 56], CRISP2 is of testicular expression [57] in differentiating spermatocytes and CRISP3 is expressed predominantly in the salivary glands, pancreas and prostate [58]. Mammalian CRISP proteins are members of a larger family of CRISP proteins found especially in reptiles with which they share the characteristic of containing 16 preserved cysteine residues. In reptiles, CRISP proteins are found in salivary secretions where they act as toxins with calcium and potassium channel blocker action [59–61]. Although the physiological functions and mechanisms of action of mammalian CRISP proteins are not proven, the high degree of identity that these proteins have with their reptilian orthologs suggests some conservation of function [62]. In humans, CRISP1 and CRISP4 are expressed in the proximal epididymis, and CRISP3 mouse was found strongly expressed in the epididymis tail and in the deferential ampoule [63].

In both humans and mice, CRISP1 is secreted in the lumen of the epididymal tubule and is found on the surface of gametes in distinct locations between the two models since in the mouse CRISP1 is located in the dorsal region of the acrosome while in the human CRISP1 is located in the post-acrosomal compartment [63]. Two populations of CRISP1 proteins are bound to gametes, a majority fraction with a labile association and a minority fraction but with solid binding. The disengagement of the labile fraction seems to be necessary for capacitation, which suggested that CRISP1 could be involved in preventing too early initiation of capacitation during transit and epididymal storage [61, 64, 65]. With respect to the minor fraction of CRISP1 firmly anchored to the gamete, it was shown that it was still present on the gamete after capacitation and migrated to the equatorial segment during the acrosomal reaction suggesting that CRISP1 could also participate in the process of interaction with the zona pellucida of the egg and more generally in gametic fusion [64, 66]. Revealing the importance of CRISP1 in the reproductive process was the observation that rats immunized with CRISP1 showed reduced fertility [67]. CRISP1 could thus be an interesting target for the development of a post-testicular contraceptive [68]. The generation of a knockout mouse model for CRISP1 further clarified the scope of CRISP1 functions. Surprisingly *crisp1*^*−/−*^ mice are fertile in natural breeding but also in in vitro fertilization with ova with intact cumulus [69]. However, sperm from Crisp1−/− animals have been shown to be less effective at in vitro fertilization of cumulus-free eggs and depellucidated eggs, indicating that CRISP1 does play a role in sperm interaction with the zona pellucida [69, 70].

Thus, with its roles as 1) an epididymal inhibitor of capacitation and 2) a modulator in primary interaction with the zona pellucida of the egg, CRISP1 offers two possibilities as a potential contraceptive target. An immunocontraceptive approach in which anti-CRISP1 antibodies could interfere with gamete recognition is thus possible. Alternatively, as the strategy chosen above for EPPIN that does not involve immune response, it will be possible to search for a pharmacological compound that may interfere with CRISP1 function in zona pellucida binding. Finally, perhaps even more promising is to interfere with the role of CRISP1 in preventing capacitation. The search for a pharmacological compound that can inhibit this decapacitating function of CRISP1 in the epididymis could lead to the production of prematurely capacitated sperm.

### P34H

P34H is a spermatic protein localized at the level of the acrosomal cap and acquired by gametes during epididymal maturation, more precisely during passage into the corpus epididymis [71, 72]. It has been suggested that P34H is involved in the interaction of sperm with the egg zona pellucida [72]. P34H has 71% identity with a tetrameric carbonyl reductase belonging to the family of short-chain dehydrogenases/reductases [72]. P34H is a post-testicular marker of fertility in men because it has been noted that the sperm P34H content in an idiopathic infertile male population was significantly lower than in the fertile control group [73, 74]. A double-blind study also showed that there was a positive correlation between the amount of P34H in male gametes and reproductive success in couples using in vitro fertilization (IVF) [75]. In order to prove the role played by this protein in reproduction its rodent ortholog (P26h: “h” for hamster) was studied further. An immunocontraceptive approach was used either with native P26h protein or with a recombinant protein coupled to a conventional carrier: Maltose Binding Protein (MBP). Male hamsters were immunized and then crossed with superovulated females. A 20 to 25% decrease in fertility was recorded following these protocols [76]. Furthermore, crossing hamster females immunized with P26h led to a significant reduction in the number of viable fetuses in those with a high blood antibody titer [77]. Thus, if P34H behaves like P26h, an immunocontraceptive strategy could eventually work. However, a search for an immunodominant epitope should be conducted in order to increase contraceptive efficacy. Surprisingly, the literature on P34H and P26h as a contraceptive target has dried up in recent years.

### SFP2

SFP2 for “sperm flagellar protein 2” is a recent candidate for the development of a post-testicular contraceptive strategy. SFP2 is one of a small group of epididymal sperm proteins identified in mice via a combined immunological and proteomic approach [78]. A human counterpart has been characterized [79]. As in previous cases the relevance of SFP2 as a contraceptive target was tested via active immunizations of male mice with two synthetic SFP2 peptides. Only one of the two peptides was able to generate high titers of anti-SFP2 antibodies that recognize the homologous protein on mouse gametes but also human and rat ortholog proteins [79]. Histological analyses of the testicles and epididymides of immunized mice did not reveal any tissue disturbances. Immune males show a very significant reduction in fertility of about 80% [79]. Incubation of spermatozoa with anti-SFP2 immune serum significantly reduces sperm mobility and viability without leading to gamete agglutination. Anti-SFP2 antibody titer in immunized animals declines 22 weeks after immunization and mouse fertility is completely restored [79]. These results are encouraging and make SFP2 a new target for the development of an immunocontraceptive approach.

### Calcineurin inhibitors

Calcineurin is a Ca^2+^- and calmodulin-dependent serine-threonine phosphatase. It is a major player in calcium signaling [80]. One of its known roles is during T-cell activation where calcineurin dephosphorylates the NFAT transcription factor (nuclear factor of activated T-cells) leading to the up-regulation of interleukine-2 [81]. Calcineurin inhibitors including cyclosporine A (CsA) and FK506 suppress T-cell activation and are at the basis of immunosuppressive strategies following organ transplantation. In animal models, it was observed in parallel that these inhibitors have detrimental effects on both spermatogenesis and epididymal sperm maturation [82, 83]. In addition, these inhibitors also impair sperm mobility and acrosome reaction [84, 85]. Interestingly, the testis expresses two calcineurins, a somatic one and a sperm-specific isoform that contains a catalytic and a regulatory subunit [86]. Mice lacking the expression of either one of these subunits were found to be infertile showing a spermatozoa phenotype of reduced motility because of a stiff sperm midpiece compartment [86]. Treatments of mice with CsA or FK506 recapitulates the KO spermatozoa phenotypes only 4 to 5 days after the treatment suggesting a post-testicular (ie: epididymal) action. Reversibility of action was observed since fertility could be recovered a week after the treatment was interrupted [86]. Human spermatozoa also exhibit these two calcineurin subunits opening the road for the development of epididymal interfering activities that could target spermatozoa within the epididymis [86].

### Agent preventing epididymal contractions

Lately, with the advancement of our general knowledge regarding the physiology of the mammalian epididymis another non-hormonal and reversible post-testicular male contraceptive strategy was brought forward. It consists in interfering with the contractile activity of the smooth muscle layer lining the cauda epididymis tubule. It was shown that the cauda epididymis is densely innervated by the sympathetic nervous system and upon ejaculation strong contractions participate in the emission of spermatozoa. Alpha_1_-adrenoceptors (α_1_-ARs) have been shown to be key actors in these contractions leading to the idea that selective α_1_-AR antagonists could be used to interfere with spermatozoa emission. Among the 3 known α1-ARs (α_1A_, α_1B_ and α_1D_) α_1A_ was shown to be the most represented in the cauda epididymis and tamsulosin (a clinically used α_1A_/α_1D_-AR antagonist) proved to be efficient in interfering with norepinephrine-induced cauda epididymal contractions in the rat [87]. This could be a promising lead providing the issue of selective and restricted administration of the antagonist agent to the cauda epididymal territory could be solved.

## Dead-end trails

### SPAM1/PH-20

The sperm adhesion molecule 1 (SPAM1) also called PH-20 is a highly conserved mammalian sperm membrane protein playing multiple roles in fertilization (reviewed in: [88]). Its localization on the sperm surface and its involvement in fertilization have made it a putative target for male immune-contraceptive strategies in both primate and non-primate species [88]. Reversible infertility was eventually achieved in both males and females guinea pigs but results in other species were less conclusive and did not lead to sterility [88]. It was assumed that these failures could be due to the absence of critical epitopes that would elicit a strong immune response. For this reason, SPAM1/PH-20 is not to date considered anymore as a promising target. In addition, although SPAM1 is of epididymal expression, it is not restricted to the epididymis since it is expressed also in the testis and other male and female accessory organs of the genital tract. With such a broad expression, collateral effects of any form of interference with SPAM1/PH-20 production or action is likely to be expected.

### SED1

SED1, standing for **S**ecreted protein showing a N-terminal domain with two **E**GF-repeats and a C-terminal region with two **D**iscoidin domains originally called p47 in porcine and harboring also various other names (MFG-E8, lactadherin, rAGS, PAS6/7 and BA-46) is a membrane component of many cells and epithelia (reviewed in: [88]). Interestingly, SED1 null-male mice were found to be subfertile in vivo harboring sperm unable to bind eggs in vitro [89]. In addition, loss of secretion of SED1/MFG-E8 from the epididymal epithelium, one of its site of expression, was associated with epididymal defects including detached epithelia and spermatic granulomas demonstrating the importance of this protein in the maintenance of the epididymis epithelium [90]. The use of SED1 antagonists as potential contraceptive agent was proposed however because of its rather crucial role on the epididymal epithelium as well as because of its rather broad roles in many other territories it was not considered as a very prominent lead.

### HE6

Human Epididymal protein 6 (HE6) also referred to as GPR64, and recently renamed ADGRG2 (standing for Adhesion G protein-coupled receptor G2) [91] is a highly epididymis-specific orphan GPCR (G-protein cupled receptor) identified some 20 years ago via differential screening of a human epididymal cDNA library [92]. Because of its belonging to this GPCR class of proteins for which a large array of pharmaceutical drugs was developed, HE6 potential as a contraceptive agent was eventually considered [93]. HE6 contraceptive potential was further confirmed by the observation that HE6 null-male mice were significantly subfertile as early as 6–9 weeks of age and sterile after 15 weeks [94]. However, failure to isolate testicular or/and epididymal ligands for HE6 put the expectation to an end.

### Epididymal oxidative stress and contraception

A recurrent factor in many male infertilities is the observation of oxidative damage to gametes. Oxidative stress and male infertility have been linked since the pioneering work of Thaddeus Man and his collaborators who observed a correlation between the peroxidized lipid content of human sperm and loss of mobility [95]. This observation was subsequently corroborated by numerous other studies [96–103]. The fact that antioxidants such as alpha-tocopherol can restore sperm mobility both in vivo and in vitro confirmed that lipid peroxidation is a major cause of loss of mobility in human gametes [104–109]. MacLeod (1943) [106] was also the first to demonstrate that incubation of spermatozoa under high oxygen stress led to rapid loss of motility and that this could be restored by the addition of catalase suggesting that hydrogen peroxide is the reactive oxygen species (ROS) involved. These results have since also been confirmed [108] and extended since lipid peroxidation induced by exposure to hydrogen peroxide not only causes a loss of gamete motility but also alters all sperm functions that depend on membrane integrity such as: fusion with the egg and the ability to trigger the acrosome reaction [109]. If we associate these observations with the high level of antioxidant protection that the epididymis provides to gametes through the presence in the fluid of primary enzymatic and non-enzymatic antioxidants [110] it logically came to mind that this aspect could perhaps be exploited for contraceptive purposes. The idea being to artificially recreate what seems to be a widespread natural cause of male infertility. Hydrogen peroxide itself or reagents that generate hydrogen peroxide when in contact with gametes may be effective contraceptive agents. Since direct exposure of spermatozoa to hydrogen peroxide disrupts their functions [111], this compound could be the basis of a topical spermostatic agent. Such a formulation would have the advantage of combining a spermicidal and a microbicidal action since vaginal sterility is naturally ensured by a low pH and by hydrogen peroxide produced by endogenous microflora.

In this attractive perspective of topical contraception via hydrogen peroxide a pitfall nevertheless appeared. To be effective, a topical spermostatic agent will have to act very rapidly on millions of spermatozoa, which hydrogen peroxide cannot do. An alternative would then be to expose the gametes to oxidative stress during epididymal descent by altering the antioxidant protection activities of the luminal environment. Such a strategy was tested in a mouse model knockout for a major primary enzymatic antioxidant (glutathione peroxidase 5, GPx5) secreted into the epididymal fluid by the epididymal head epithelium [112]. The lowest epididymal antioxidant protection in *gpx5*^*−/−*^ mice led to oxidative damage to spermatozoa, mainly visible at the sperm nucleus [113]. Such damage does not affect fertilization but led to defects in embryonic development when older *gpx5*^*−/−*^ males were crossed with wild females [113]. This result highlights another pitfall of a pro-oxidant epididymal contraceptive approach which is that oxidative stress is associated with damage to sperm DNA with possible consequences on embryonic development and possible transmission of genetic abnormalities to offspring [113, 114]. Echoing these observations in the mouse model, it should be noted that high levels of damage to sperm DNA have been linked in humans to: pre-implantation embryonic development abnormalities, increased rates of early abortion and increased morbidity in offspring illustrated by an increased frequency of dominant monogenic pathologies, infertility and cancers [114]. In the fairly recent past, two advances had made it possible to understand certain spontaneous male infertilities: deletions of the Y chromosome and the observation, already mentioned above, that many cases of male infertility were associated with oxidative damage to spermatozoa. Although the mechanisms responsible for spontaneous deletions of the Y chromosome in infertile men are not yet resolved, two explanations are put forward. One suggestion is that there would be intra-chromosomal recombination events in the father’s germ line involving large blocks of repeated sequences [115, 116]. Another explanation would be that these recombination events would occur after fertilization when the fertilized egg tries to repair the damage to the paternal nucleus. In the first suggestion, the deletions of the Y chromosome would be detectable in the father’s gametes, while in the second suggestion, the deletions would be visible only in the male offspring, the father’s spermatozoa then showing only a high rate of DNA damage. Such damage to sperm DNA is very widespread in men and closely correlated with infertility. The ethiology of this damage is associated with oxidative stress in the germline [116]. Thus, the main causes of spontaneous male infertility: deletion of the Y chromosome and oxidative damage to the gametes nucleus could somehow be linked [116].

In light of these developments and the consequences that the induction of epididymal oxidative stress could have on sperm cells, such a contraceptive approach has been abandoned.

### Another abandoned lead: interfering with the sperm’s ability to regulate their volume

One of the earliest observations of post-testicular infertility was the “Dag” phenotype named after the affected Jersey bull [117]. The spermatozoa of this animal exhibited a characteristic 180° angulation of the flagella at the junction of the intermediate piece and the main piece. Such a phenotype has subsequently been found in many infertile bulls of different breeds as well as boars, dogs and stallions [117]. The spermatic phenotype was quite rapidly associated with epididymal dysfunctions and was the first demonstration that one or more alterations in epididymal maturation could result in infertility. Some twenty years later, a similar phenotype (angulated spermatozoa + infertility) was found in several lines of transgenic mice knockout for genes expressed in the proximal part of the epididymis head, the initial segment [118]. Thus, a dysfunction in the initial segment of the epididymis head resulted in infertility associated with functional deficiencies of the spermatozoa. Exploration of these transgenic models has shown that flagellar angulation results from the inability of sperm to regulate their volume in hypotonic situations such as during ejaculation and when they arrive in the female genital tract. The resulting swelling causes membrane tensions that generate angulation [119].

Spermatozoa, like any somatic cell, regulate their volume by the efflux of osmolytes and water associated with them. Thus, if in the models mentioned above the spermatozoa are no longer able to regulate their volume in a hypotonic situation, it is either because they are exposed during their transit through the deficient epididymis of these transgenic animals to a hypotonic situation inducing the loss of osmolytes, or because they have a lower supply of these osmolytes. The osmolarity of the epididymal fluid was not found to differ between the control animals and the transgenic animals. However, the content of different classical osmolytes (carnitine, taurine, myo-inositol, glutamate) in the spermatozoa of transgenic models was found to be reduced [120–122]. Thus, the osmolyte supplies provided when the gametes descend into the epididymal tubule are reduced in transgenic models. The idea then emerged that if one could interfere with the epididymal contribution of osmolytes to gametes during their epididymal maturation or block the release of these osmolytes in the hypotonic situations they will face, one could possibly approach the “DAG” context or that encountered in transgenic mouse models and thus induce sterility [123]. In theory, this can be achieved by 1) blocking the epididymal secretion of osmolytes, 2) blocking the import of these osmolytes into the gametes or 3) blocking the efflux of these osmolytes out of the gametes in hypotonic post-ejaculation situations. Large-scale transcriptome and epididymal proteome analyses did not identify enzymes and transporters responsible for osmolyte secretion that were specific to the epididymis and could have been pharmacologically targeted. On the other hand, concerning the efflux of sperm osmolytes in hypotonic situations, several channels that can mediate the export of osmolytes have been found on spermatozoa of various species including humans [123]. Despite the fact that specific inhibitors of these channels exist and could thus be good candidates for post-testicular contraception, none of these inhibitors have irreversible or sufficiently long-lasting effects to meet the required efficacy criterion. This avenue of research, which originally seemed promising, is no longer in the spotlight today.

## Conclusions

Although the idea of targeting the epididymis and the changes it induces in male gametes for the development of new post-testicular and non-hormonal contraceptive strategies is particularly attractive, it will still be a few years before such a contraceptive agent is on the market. The idea appeared very early on and is based on solid foundations that are essentially:

- greater safety due to the absence of interference with testicular function and complex hormonal regulation of the hypothalamic-gonadal axis,
- the possibility of faster action in the acquisition of infertility and reversion of infertility compared to spermatogenesis blockage,
- easier access to oral contraceptives due to the closer relationship between epididymis lumen and the blood compartment. The latter issue is interesting because it may eventually result in a decrease in the need for contraceptive agents to achieve efficacy, which may limit the occurrence of potentially harmful side effects.

However, despite these opportunities, the development of an epididymal contraceptive continues to face challenges. Among them, the lack of basic knowledge on the physiology of the mammalian epididymis, the critical mass of the international scientific community involved in this field and, consequently, the lack of academic and private funding to support the efforts of scientists and clinicians have considerably hindered the advancement of knowledge in this sector [124]. In addition, a contraceptive compound targeting sperm in the epididymal fluid may potentially reach the female reproductive system during intercourse through seminal plasma, raising safety concerns not only for men but also for their female partners. This aspect should be the subject of parallel studies and will certainly limit regulatory approval so that this contraceptive can be quickly brought to market.

These last 10 years have, however, brought many new developments that have led to some very promising leads. This has been made possible by the arrival of large-scale investigative technologies that have made it possible to identify the transcriptome and epididymal proteome of mammals, revealing a range of potential contraceptive targets that meet the criteria of specific expression and possible pharmacological targeting. Recent progress has also been made possible by the impetus given by the establishment of international research networks in a unique public-private partnership. For the record, the AMPPA network “Applied Molecular Pharmacology for Post-testicular Activity” supported from 1999 to 2007 by the Rockefeller Foundation (New York, USA), the ESRF “Ernst Schering Research Foundation” (Berlin, Germany) and CONRAD “Contraceptive Research and Development” (New York, USA) greatly stimulated and facilitated interactions between researchers interested in the epididymis and its functions as a contraceptive target. It is regrettable that such actions have not been more sustainable and that the pharmaceutical industry has completely turned its back on this sector. Yet, global population growth, the alarming number of unwanted pregnancies worldwide, men’s desire to take a more active role and share control over their fertility and family planning [3] argue for an expansion of male contraceptive supply.

In view of the latest developments presented above, it seems that immunocontraception with a spermatic target for post-testicular acquisition is one of the most popular strategies. Contraceptive vaccines have been tested for many years and at several levels, since they can target gamete production (LH/GnRH vaccines), gamete functions (vaccines against sperm antigens or against egg zona pellucida proteins) or indirectly fertilized zygote (hCG vaccine) (for review see: [125, 126]). Nevertheless, some pitfalls remain regarding the inter-individual variability of immune responses requiring the development of more elaborate approaches. Solutions are in line with the focus on: the selection of more immune epitopes on targeted proteins, the use of combined targets, the development of synthetic antibodies of the ScFv “single chain variable antibody fragment” type [125, 126] which, lacking the constant fragment, minimize certain slopes of the immune antibody-dependent response.

Direct pharmacological approaches that would aim to inhibit epididymal functions so as to render gametes non-fertilizing are still pending. Only EPPIN, discussed above, offers an interesting alternative to immunocontraception to date since organic compounds that have the ability to block one of the action sites of the protein (semenogelin binding) resulting in inhibition of gamete mobility are being studied [51].

## Abbreviations

AEG protein: Acidic Epididymal Glycoprotein
AMPPA: Applied Molecular Pharmacology for Post-testicular Activity
CLU: Clusterin
CONRAD: CONtraceptive Research and Development
CRISP: Cysteine-Rich Secretory Protein
CsA: Cyclosporine
DE protein: Distal Epididymal protein
EPPIN: EPididymal Protease INhibitor
ESRF: Ersnt Schering Research Foundation
FK506: Fujimycine = Tacrolimus = Calcineurin inhibitor
GnRH: Gonadotropin Releasing Hormone
GPx5: Glutathione Peroxidase 5
hCG: Human Chorionic Gonadotropin
HE6: Human Epididymal protein 6
IVF: In Vitro Fertilization
KO: knock-Out
LH: Luteinizing Hormone
LTF: Lactotransferine
MBP: Maltose Binding Protein
NFAT: Nuclear Factor of Activated T-cells
P26h: 26 kilo Dalton hamster sperm protein
P34h: 24 kilo Dalton human sperm protein
PSA: Prostate Specific Antigen
ROS: Reactive Oxygen Species
ScFv: Single-chain Variable fragment
SED1: **S**ecreted protein showing a N-terminal domain with two **E**GF-repeats and a C-terminal region with two **D**iscoidin domains
SFP2: Sperm Flagellar Protein 2
SPAM1: Sperm Adhesion Molecule 1
SPINLW1: Serine Peptidase INhibitor-Like protein with Kunitz and WAP domains 1
TPX1: Testicular Protein X1
WAP-type 4-DSC: Whey Acidic Proteins type 4-disulfide core
